# The iTClamp in the treatment of prehospital craniomaxillofacial injury: a case series study

**DOI:** 10.5249/jivr.v11i1.917

**Published:** 2019-01

**Authors:** Jessica L. Mckee, Ian A. Mckee, Chad G. Ball, Edward Tan, Alan Moloff, Paul McBeth, Anthony LaPorta, Brad Bennett, Dennis Filips, Carrie Teicher, Andrew W. Kirkpatrick

**Affiliations:** ^*a*^Department of Surgery, Foothills Medical Center, University of Calgary, Calgary, Canada.; ^*b*^Department of Fire, City of Edmonton, Edmonton, Alberta, Canada.; ^*c*^Regional Trauma Services and the Departments of Surgery, and Critical Care Medicine, University of Calgary, Calgary, Alberta, Canada.; ^*d*^Alberta Health Services, Foothills Medical Centre, Calgary, AB, Canada.; ^*e*^Royal Netherlands Army, Radboud University Medical Center, The Netherlands.; ^*f*^Pyng Medical, Richmond, BC, Canada.; ^*g*^Rocky Vista University, Parker, CO, USA.; ^*h*^Department of Military & Emergency Medicine, Edward Hebert School of Medicine, USA.; ^*i*^Innovative Trauma Care, Edmonton, AB, Canada.; ^*j*^Medecins Sans Frontiers / Doctors Without Borders, New York, NY, USA.; ^*k*^Canadian Forces Health Services, Canada.

**Keywords:** iTClamp, Prehospital, Craniomaxillo facial

## Abstract

**Background:**

Craniomaxillofacial (CMF) injuries are very common in both civilian and military settings. Nearly half of all civilian trauma incidents include a scalp laceration and historical rates of CMF battle injuries increased from 16%-21% to 42.2%. The scalp is highly vascular tissue and uncontrolled bleeding can lead to hypotension, shock and death. Therefore, enabling on-scene providers, both military and civilian, to immediately manage scalp and face lacerations, in a manner that allows them to still function in a tactical way, offers operational advantages. This case series examines how effectively a wound-clamp (iTClamp) controlled bleeding from CMF injuries pre-hospital environment.

**Methods:**

The use of the iTClamp for CMF (scalp and face laceration) was extracted from iTrauma Care’s post market surveillance database. Data was reviewed and a descriptive analysis was applied.

**Results:**

216 civilian cases of iTClamp use were reported to iTrauma Care. Of the 216 cases, 37% (n=80) were for control of CMF hemorrhage (94% scalp and 6% face). Falls (n=24) and MVC (n=25) accounted for 61% of the mechanism of injury. Blunt accounted for 66% (n=53), penetrating 16% (n=13) and unknown 18% (n=14). Adequate hemorrhage control was reported in 87.5% (n=70) of cases, three respondents reported inadequate hemorrhage control and in seven cases hemorrhage control was not reported. Direct pressure and packing was abandoned in favor of the iTClamp in 27.5% (n=22) of cases.

**Conclusions:**

CMF injuries are common in both civilian and military settings. Current options like direct manual pressure (DMP) often do not work well, are formidable to maintain on long transports and Raney clips are a historical suggestion. The iTClamp offers a new option for control of external hemorrhage from open wounds within compressible zones.

## Introduction

Craniomaxillofacial (CMF) injuries are very common in both civilian and military settings. Nearly half of all civilian trauma incidents include a scalp laceration.^1^ Rates of CMF battle injuries have risen from 16%^2^ to 42.2%, with more than 50% of all battle injury patients in the Navy-Marine Corps Combat Trauma Registry incurring one or more injuries to the head, face, or neck.^3^ CMF injuries may involve almost all organ systems and impair many critical functions such as ventilation, cognition and vision. But among the multitude of important structures and functions at risk during CMF injury, bleeding is the most notable concern. As the CMF region contains major vascular structures and is generally composed of highly vascular tissue, injuries can lead to uncontrolled bleeding, hypotension, shock and death.^4-6^ Even seemingly innocuous injuries can lead to higher than anticipated blood loss.^7^In order to prioritize the immediate resuscitative care, typically initialized in the field, providers should choose to apply the MARCHE priorities principle (massive bleeding, airway, respirations, circulation, hypothermia, everything else /evacuation)^7^ and obtain hemostasis at the earliest opportunity as the highest priority in treatment and stabilization. 

Many lessons in hemorrhage control have been learned in the last decade and half of conflict. For example, focused efforts on preventing extremity exsanguination have been remarkably effective in reducing unnecessary death. Frequent emergency tourniquet application has reduced death 85% from 23.3 deaths per year to 3.5 death per year.^8^ However, tourniquets are not applicable to CMJ injuries and CMF injuries are challenging to treat in the field. These injuries can be hard to access, treatment techniques are cumbersome, dressings are hard to apply, migrate and consistent direct pressure is demanding to maintain.^3,4,7,9^ These challenges have led to the need to examine other rapidly deployable hemorrhage control options. Raney clips are a standard operative neurosurgical tool, with the potential for use outside the OR. There potential in scalp injuries was examined as long as 25 years ago^10^but aside from a small number of Emergency Departments (ED’s) and limited use in the field, Raney clips have not gained wide-spread usage,^4^ presumably as they require a formal application instrument to apply and they do not seal the wound only the wounds edges. 

Therefore, enabling on-scene providers, both military and civilian, to immediately manage CMF injury in a manner that allows them to still function in a tactical way, offers operational advantages. This case series thus examines effectiveness of a novel ergonomic topical wound-clamp, the iTClamp for in controlled bleeding from the CMF region potentially enabling first responders to effectively stop CMF bleeding in the pre-hospital environment.

## Methods 

**Figure 1:**
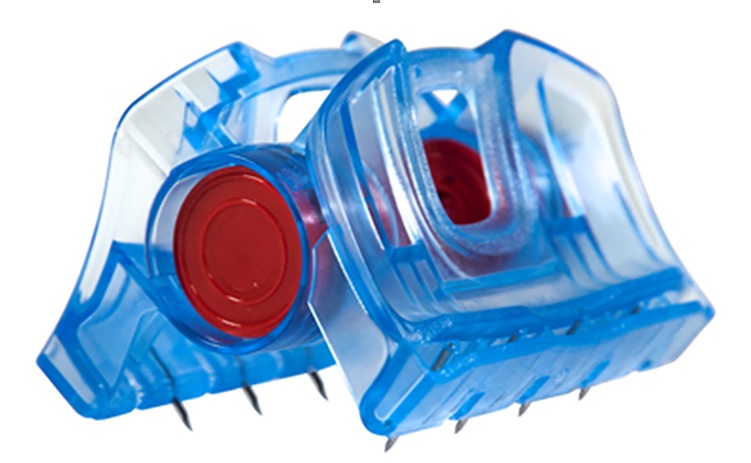
Version 2 of the iTClamp is displayed in the image above. This is the current version that is available. In the patient images version 1 of the iTClamp is used. However, the two versions are substantially equivalent and function the same.

The iTClamp (Innovative Trauma Care Inc, San Antonio, Tx) is a self-locking hemostatic clamp with eight needles that penetrate the skin to evert the skin edges between pressure bars. Pressure is evenly distributed across the bars, which seal the skin over the wound. This action stops the bleeding by creating a temporary contained hematoma, until surgical repair (Figure 1). As part of FDA regulation, Innovative Trauma Care (San Antonio, Tx) tracks iTClamp use for post market surveillance and cases are submitted voluntarily. The use of the iTClamp for treatment of CMF injuries was extracted from Innovative Trauma Care’s post market surveillance database. Data was reviewed and a descriptive analysis was applied. Data analysis was completed using SPSS (IBM Corp. Released 2013. IBM SPSS Statistics for Windows, Version 22.0. Armonk, NY: IBM Corp.) 

## Results

From April 2013 – December 2015, 216 civilian cases of iTClamp use were reported to Innovative Trauma Care. Of the 216 cases, 37% (n=80) were for control of CMF hemorrhage (94% scalp and 6% face). Males accounted for 57.5% (n=46) of cases, females 23.8% (n=19) and in 15 cases (18.7%) gender was not reported. The mean patient age was 53.3 years ± 22.9 years with a minimum age of 16 years and a maximum age of 95 years. Sixty-one percent of cases were reported from Europe, 35% from North America and 4% from other countries. Falls (n=24) and motor vehicle crashes (MVC) (n=25) accounted for 61% of the mechanisms of injury, with the remaining 39% of mechanisms reported in Figure 1. Blunt injury accounted for 66% (n=53), penetrating injury 16% (n=13) and 18% (n=14) were unknown. Emergency Medical Services (EMS) reported the most use (n=50, 62.5%), followed by Air Transport (n=14, 17.5%) and ED (n=10, 12.5%); in six cases (7.5%) the environment of application was not reported. An average of 1.2 ± 0.5 devices was used per patient. The minimum number of devices used per patient was one and there was a maximum of four devices used. Adequate hemorrhage control was reported in 87.5% (n=70) of cases, three respondents (3.75%) reported inadequate hemorrhage control and in seven cases (8.75%) hemorrhage control was not reported. For the three cases of inadequate hemorrhage control, two respondents reported that the bleeding was controlled but did not stop and in the third case the patients skin was frail and it could not hold the iTClamp. Seven respondents (9%) reported the need to reapply the device with six (86%) reporting adequate hemorrhage control on the second application. The seventh case where hemorrhage control was not obtained after reapplication is the case of the patient with frail skin mentioned above. 

Direct pressure and packing were abandoned in favor of the iTClamp in 27.5% (n=22) of cases and in the patient with frail skin the iTClamp were abandoned in favor of gauze and direct pressure. The iTClamp was applied to three patients that were anti-coagulated. Adequate hemorrhage control was reported for one, and not reported for the other two. Application-time was under a minute in 41.3% (n=33) of cases, with only 2.4% (n=2) requiring over one minute of application time and in 56.3% (n=45) application time was not reported. We discuss a few of the cases below in more detail.

**Figure 2:**
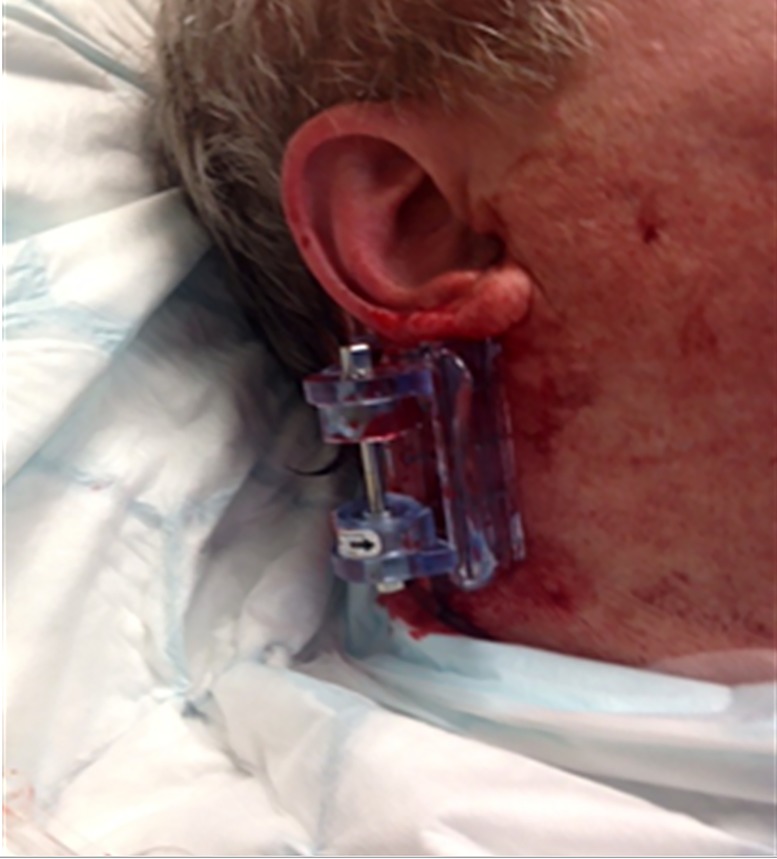
Case 1 iTClamp application to the neck. Following a post-operative bleed from the surgical removal of a basal cell carcinoma in the neck.

**Case 1: **This was an arterial bleed two weeks after the surgical removal of a basal cell carcinoma in the neck. The patient started to bleed from the incision and had lost half a liter of blood and had soaked through 4 gauze pads before reaching the hospital. On arrival to hospital the patient 's bleeding was still not controlled. The iTClamp was applied to the neck (Figure 2) in hospital and remained in place for 25 minutes. When the iTClamp was removed to suture, the bleeding had stopped.

**Figure 3:**
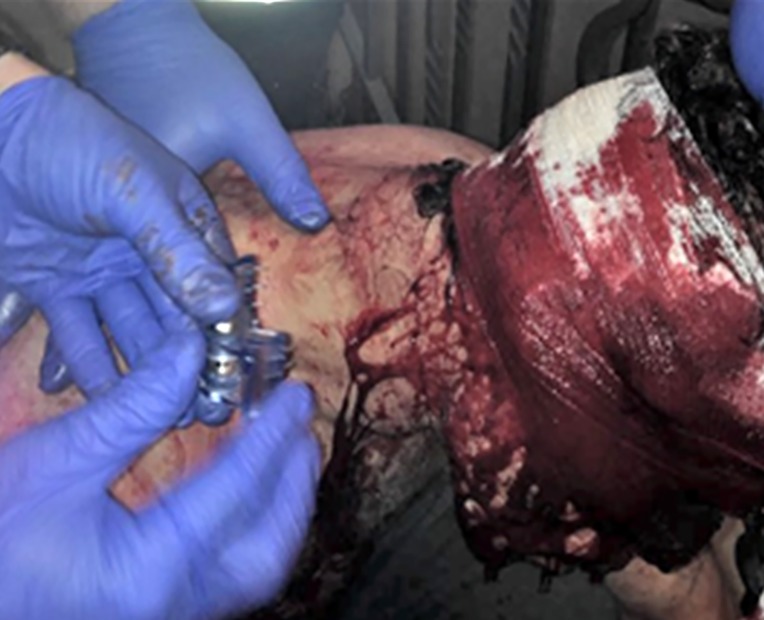
Case 2: Stab wound to the temporal area This patient was stabbed twice once in the temporal area and once in the neck following an altercation. This image is pre iTClamp application but post gauze application. This image demonstrates the amount of blood loss and wound severity.

**Figure 4:**
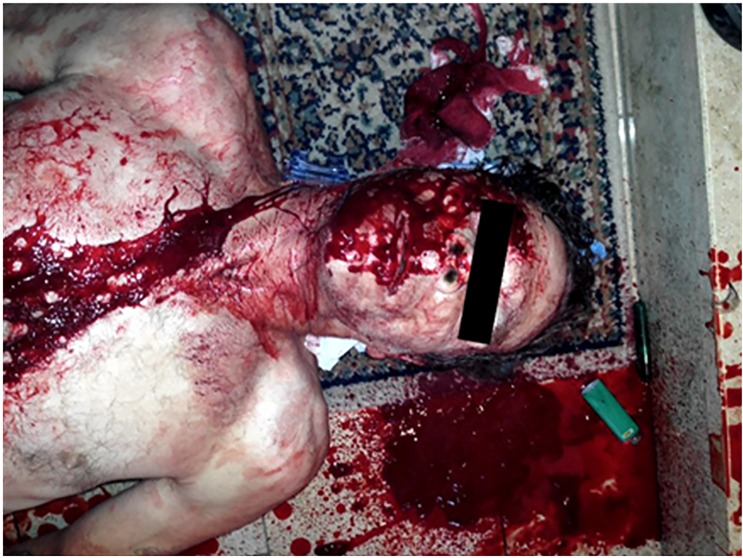
Case 2: Scene shot post iTClamp application This is an image of the scene after iTClamp application to the patients temporal region and neck. The bleeding was now under control. It was approximated that the patient lost 1.5 liters of blood on scene from the two stab wounds he sustained.

**Figure 5:**
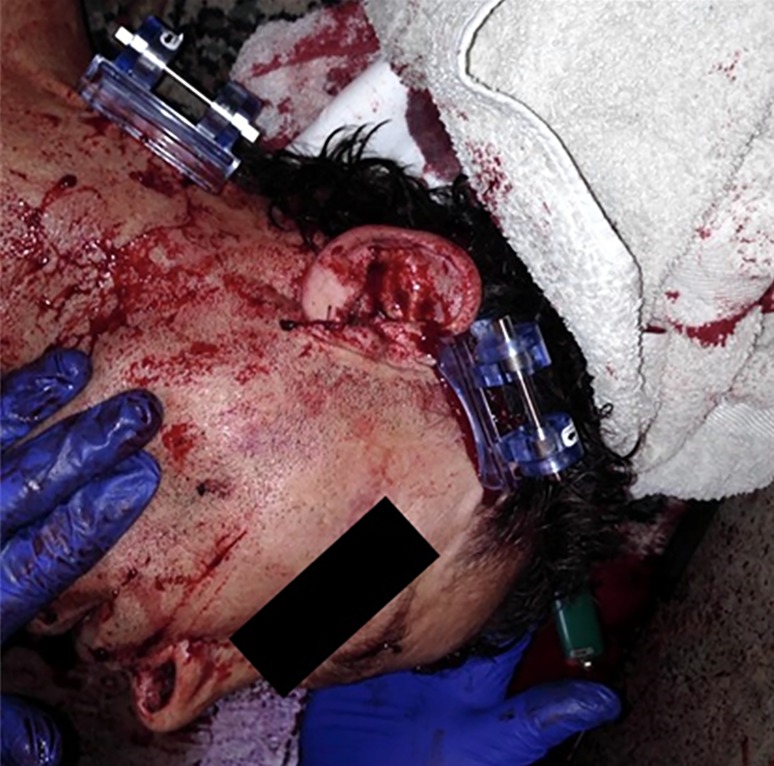
Case 2: iTClamp application One iTClamp was applied to each of the stab wounds (one to the temporal region and one to the neck) to stop the bleeding. First responders reported no issues with airway management.

**Case 2:** A 54-year-old man was assaulted with a knife and sustained two stab wounds. The first stab wound was to the right side of neck and a second stab wound was to the right temporal area (Figure 3). The patient lost approximately 1.5 liters of blood on the scene (Figure 4). A single iTClamp was applied to each wound and the bleeding stopped immediately (Figure 5).

There were issues with airway management due to the neck injury. It was easy to intubate the patient before he was transferred to the operating theater. At the theater they removed the iTClamp from the neck and the bleeding started spurting again. They replaced the iTClamp and started carefully with the wound treatment and definitive care. 

**Figure 6:**
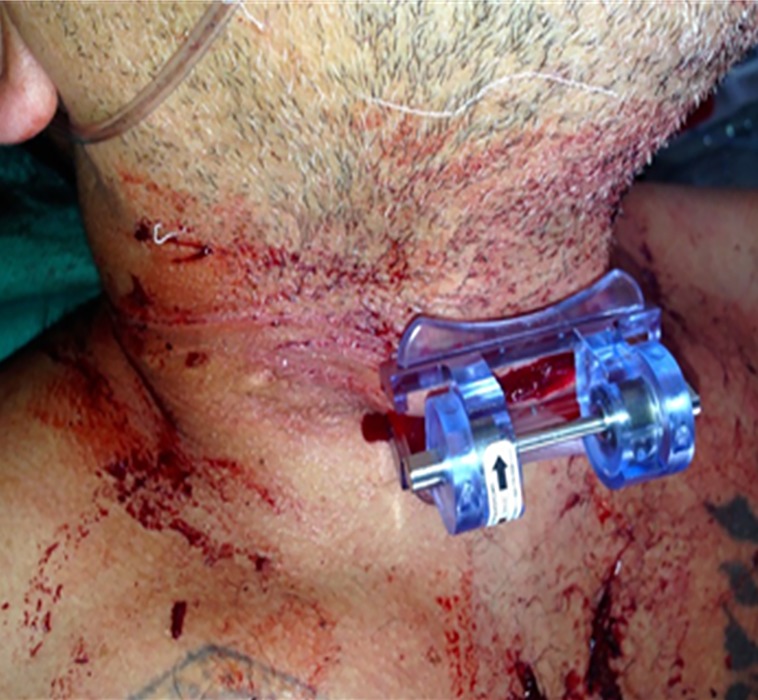
Case 3: iTClamp neck application following a self-inflicted injury Post iTClamp application to the neck following a series of self-inflicted injuries. No issues of airway management were reported.

**Case 3:** This was a 47-year-old male patient who before being arrested by police took 50 tablets of clonazepam (benzodiazepin) and escaped. While on the run he caused a car accident and was admitted to hospital. After two days he was discharged home from hospital where he tried to take his own life with a knife and broken glass bottle. When paramedics arrived on the scene, no first aid had been given by the wife. The patient had 19 lacerations in total with two severe lacerations, including one slash wound to the lower larynx with external hemorrhage. The physician on scene applied one iTClamp to the neck to stop the bleeding (Figure 6).

## Discussion

The prevalence of CMF injuries is significant in both the civilian and military setting.^1,3^ However, despite the prevalence rate and negative sequel associated with uncontrolled CMF hemorrhage there is still a tendency to ignore these injuries in favor of the completion of the primary and secondary surveies.^4^ This tendency highlights the notion that care providers continue to underestimate the amount of blood that can be lost from the scalp.^4^ During mass casualty, where a diminution in the quality of trauma care is experienced,^11^ discounting CMF hemorrhage could be especially prominent. The current standard of applying direct pressure may not be manageable during mass casualty or even when a single patient has multiple injuries that require attention.^7^ Other potential techniques such as utilizing Raney clips,^4^ or lassoing the artery in order to ligate the vessel,^7^ have been previously suggested as an alternative to direct pressure. The value in these alternatives is that they allow the care providers to be hands free to deal with other patients, other issues or even transport to radiology.^5^ However, Raney clips have experienced limited up-take, perhaps because of the restricted scope of use and need for an applicator. The ability to suture, specifically to lasso and ligate a vessel, is something that requires a great deal of skill/practice, and has been shown to extremely challenging or even impossible for non-surgical first responders.^12^ The increasing rate of CMF injuries, particularly on the battle field cannot be ignored. Organizers need to be cognoscente of the growth of CMF injuries and continue to be vigilant in finding new strategies and equipment that can prevent and treat these injuries^3,13^ both on the battlefield and in civilian practice. 

Such an option may be the iTClamp, a temporary wound closure device, which controls external hemorrhage from open wounds within compressible zones. Pre-clinical papers have demonstrated that the iTClamp provides superior hemorrhage control to packing alone,^14^ reduces fluid loss,^15^ is not impacted by movement,^15^ improves survival,^16^ does not cause further skin damage,^17^ is superior to suturing in an emergent situation especially for non-surgical first responders^12^ and can be used even with limited medical knowledge;^17,18^ findings that have been supported during clinical assessment.^19-23^

The head, face and neck are only 12% of the bodies surface area exposed during combat;^24^ however, this area is not well protected partially because any obstruction in this zone can limit senses that are vital in hostile environments.^3^ Combine this with the increase use of Improvised Explosive Devices (IED’s) by enemy combatants and it’s no surprise that the CMF region is experiencing disproportionally more injuries leading to death.^13^ This increase in potentially fatal hemorrhage injuries means that a device, such as the iTClamp, can stop the bleeding, and allows providers to be hands free has operational merit. 

Limitations of this study is that it is a retrospective review of a voluntary data submission which may produce a bias sample.

## Conclusion

It has been demonstrated in the literature that CMF injuries are common in both the civilian and military realms. Current options like direct manual pressure (DMP) that often do not work well, are formidable to maintain on long transports and Raney clips are a historical suggestion. The iTClamp offers a new option for control of external hemorrhage from open wounds within compressible zones. This case series suggests that the iTClamp may be considered as an alternative or adjunct to DMP for use in controlling exsanguination from CMF injuries in both the civilian and military settings. 

**Competing Interest:** Jessica Mckee is the Clinical Director of Innovative Trauma Care, the company that funded this study and manufactures and distributes the iTClamp, one of the devices tested in this study. Jessica Mckee has had her travel covered by Innovative Trauma Care as part of her position with the company and is entitled to stock options. Dennis Filips is the Chief Medical Officer of Innovative Trauma Care, the company that funded this study and manufactures and distributes the iTClamp, one of the devices tested in this study. Dennis Filips sits on the Board for Innovative Trauma Care, has stock in the company and has his name on several patents with Innovative Trauma Care. Dennis Filips has received an honorarium to speak for the University Of Calgary De-partment Of Surgery and his travel is covered by Inno-vative Trauma Care as part of his position with the company. Ian Mckee is the husband of Jessica Mckee the Clinical Director for Innovative Trauma Care. Ian is also a contract educator for Innovative Trauma Care and the company has paid for his travel when it is a result of his position with the company. Major Andrew W. Kirkpatrick has been paid a consulting fee and travel compensation from Innovative Trauma Care. An-drew Kirkpatrick has also consulted for Acelity and Cook Medical, Cook Medical has also paid for his trav-el on other projects. No other authors have anything to declare.
